# Supplementary material for: Workplace stress and associated factors among vehicle repair workers in Hawassa City, Southern Ethiopia
Source: PLoS One. 2021 Apr 5;16(4):e0249640. doi: 10.1371/journal.pone.0249640 (PMC8021151; doi:10.1371/journal.pone.0249640)
Supplement: S1 File — (DOCX) [file pone.0249640.s001.docx]

# English version verbal consent form

How are you? My name is ………………………………….. I am here on behalf of **Hailemichael Mulugeta**, is a researcher and conducting research on the prevalence of self-reported workplace stress and associated factors among vehicle repair workers in Hawassa City, South Ethiopia. He has received ethical clearance from Hawassa University College of Medicine and Health Sciences Institutional Review Board. Letter of the permission was obtained from concerned Governmental offices. The main activities are measuring height and weight, collecting information about your sociodemographic character, personal characteristics and symptoms or pain in nine body regions; and work environment characteristics. We are very interested in your experiences and your point of view. We will start by asking your willingness to participate in the study and will clearly explain to you the objective, benefit, and risks of the study to get your consent. Questions are simple and related to what you know in your daily activities. Please feel free to speak; your or company name or anything that could identify you personally will not be used in any official reports or presentations.

Then, kindly respond accordingly:

1. If yes, continue the interview.

2. If no, skip to the next participant by writing the reasons for his/her refusal.

**Informed consent Certified by:**

Participant code: --------------

Interviewer name-----------------------------signature---------------

Date of interview-----------------Time started--------------------Time completed-------

**Result of the interview:**

1. Completed 2. Partially completed 3. Refused

**Reasons for his/her refusal**: ----------------------------------------------------------------------------------------------------------------------------------------------------------------------------------------------------

**Checked by:**

Supervisor Name--------------------------signature----------------Date------

Questionnaire identification number --------------

# English version of the questionnaire

**Instruction**: Interviews should be conducted in a private space or room. Then allow the participant to feel free, ask and circle or write the answer. If the answer is other than your choice, please write it on the black space.

**Date of interview------------- Questionnaire identification number** **----------------**

**Section One: - *Socio-demographic characteristics of respondents***

| **No** | **Questions** | | **Responses** | | **Skip** |
| --- | --- | --- | --- | --- | --- |
| **101** | Sex | | 1.Male  2.Female | |  |
| **102** | Age in completed years (NB. Please round up if the month is Known) | | _________________ | |  |
| **103** | What is your marital status? | | 1. Married 4. Single  2.Divorced 5. Cohabited  3.Widowed 6. Other__________ | |  |
| **104** | What is your educational status? | 1. Unable to read and write 2. Read and write only 3. Primary (grade 1-8) | 4.Secondary (grade 9-12)   1. College/ TVET | 1. Degree and above |  |
| **105** | What is your employment status? | | 1. Permanent worker 2. Temporary/Contract worker | |  |
| **106** | What is your job position? | | 1. Senior Mechanic 2. Assistant/ Apprentice/Helper | |  |
| **107** | How many years do you work in vehicle repair? | | ________ Years _______Months | |  |
| **108** | Would you tell me your monthly net income in Ethiopian Birr? | | - - - 1. <2500       2. 2500-5000   3. 5001-7500  4. >7500 | |  |

**Section two:** Personal related information

| **No** | **Questions** | **Responses** | **Skip** |
| --- | --- | --- | --- |
| **201** | Weight in Kg | _________kg |  |
| **202** | Height in centimeter | _________ cm |  |
| **203** | What type of activity do you after leaving this work? | 1. The same type 2. Watching movies/ Reading 3. Playing sports/games 4. Other_________ |  |
| **204** | Do you practice physical exercise includes any kind of sports at least two times per week with a duration of 30 minutes? | 1. Yes 2. No |  |
| **205** | Do you smoke Tobacco/cigarette at least one stick of cigarette a day? | 1. Yes 2. No |  |
| **206** | Do you drink any kind of alcohol at least twice a week? | 1. Yes 2. No |  |
| **207** | Have you ever chewed khat? | 1. Yes 2. No | 301 |
| **208** | If yes, have you chewed Khat in the last 30 days? | 1. Yes 2. No |  |

***Section three* –**Work environment related information

| **No** | **Questions** | **Response** | | **Skip** |
| --- | --- | --- | --- | --- |
| **301** | Have you ever taken professional training in car repair and maintenance? | 1. Yes 2. No | |  |
| **302** | Have you ever taken occupational health and safety training? | 1. Yes 2. No | |  |
| **303** | What type of workshop do you work for? | 1.Private  2.Government | |  |
| **304** | What type of vehicle do you engage in repair and maintenance? | 1. Sedan/ Saloon car 5. Minibus/Minivan 2. Pick up/ Light truck 6. SUV/4WD 3. Truck/ Large 4. Bus | |  |
| **305** | What is your major job category or responsibility?  (Circle all if there is more than one responsibility/ Job category) | 1. Mechanical Repair *(Service and repair, engine tune-ups, oil changes replacing filters, wheel service, alignment, tire rotation, wheel balancing, and changes, etc…)* 2. Electric Repair 3. Panel beating /Body Wielding *(Bat Lamera)* 4. Spray painting 5. Other…… | |  |
| **306** | How many days do you spend on work per week? | | __________Days |  |
| **307** | How many hours do you spend on your work per day? | | __________Hours |  |
| **308** | What ground/floor type do you have at your work place? | | 1. Concrete 2. Gravel/ Pebbles 3. No ground covers 4. Others specify_______ |  |
| **309** | What is your most commonly adopted posture to caried the job? | | 1. Standing 2. Sitting 3. Kneeling 4. Bending 5. Squatting 6. Lying on the ground 7. Reaching overhead |  |
| **310** | Do you often, in your work, lift, push, pull carry or move very heavy loads (exceeding 20kgs) without peoples help or an assistive tool? | | 1. Yes 2. No |  |
| **311** | Do you work at inadequate workspace to do your work properly? | | 1. Yes 2. No |  |
| **312** | Is there shortage of staff than the need? | | 1. Yes 2. No |  |

***Section four:*** *Questions* to measure job stress

| **No.** | **Questions /variables** | **Job stress score** | | | | |
| --- | --- | --- | --- | --- | --- | --- |
|  |  | Never | Rarely | Sometimes | Often | Very often |
| **401** | Conditions at work are unpleasant or  sometimes even unsafe | 1 | 2 | 3 | 4 | 5 |
| **402** | I feel that my job is negatively affecting my physical or emotional wellbeing | 1 | 2 | 3 | 4 | 5 |
| **403** | I have too much work to do and/or too many  unreasonable deadlines | 1 | 2 | 3 | 4 | 5 |
| **404** | I find it difficult to express my opinion or feelings about my job conditions to my superiors. | 1 | 2 | 3 | 4 | 5 |
| **405** | I feel that job pressures interfere with my family or personal life. | 1 | 2 | 3 | 4 | 5 |
| **406** | I have adequate control or input over my work duties. | 5 | 4 | 3 | 2 | 1 |
| **407** | I receive appropriate recognition or rewards for good performance. | 5 | 4 | 3 | 2 | 1 |
| **408** | I am able to utilize my skills and talents to the fullest extent at work | 5 | 4 | 3 | 2 | 1 |
|  | Total score Result=_­ | | | | | |

***Section five:*** *Questions to measure job satisfaction*

| **No.** | **Questions /variables** | **Job satisfaction score** | | | | |
| --- | --- | --- | --- | --- | --- | --- |
|  |  | Strongly Disagree | Disagree | Do not know | Agree | Strongly  satisfied |
| **501** | I receive recognition for a job well done | 1 | 2 | 3 | 4 | 5 |
| **502** | I feel close to the people at  Work | 1 | 2 | 3 | 4 | 5 |
| **503** | I feel good about working at this Company | 1 | 2 | 3 | 4 | 5 |
| **504** | I feel secure about my job | 1 | 2 | 3 | 4 | 5 |
| **505** | I believe management is concerned about me | 1 | 2 | 3 | 4 | 5 |
| **506** | On the whole, I believe work is  good for my physical health | 1 | 2 | 3 | 4 | 5 |
| **507** | My wages are good | 1 | 2 | 3 | 4 | 5 |
| **508** | All my talents and skills are used at work. | 1 | 2 | 3 | 4 | 5 |
| **509** | I get along with my supervisors | 1 | 2 | 3 | 4 | 5 |
| **510** | I feel good about my job | 1 | 2 | 3 | 4 | 5 |
|  | Total score Result=_­_________________ | | | | | |

**Instruction to WMSD information**

**Questioners to assess WMSD in the neck, shoulder, upper back, lower back, hip /thigh, knee/leg, ankle/foot and wrist /hand. The diagram below shows the approximate position of the body parts referred to in the questionnaire.**

**
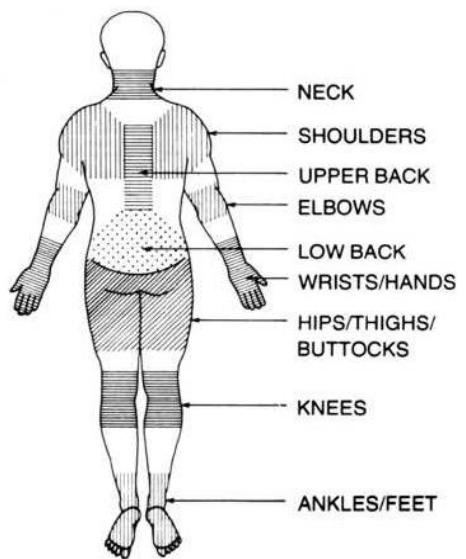
**

Have you had trouble at any time during the last 12 months (ache, discomfort and pain) lasting 2-3 workdays that resulted in either:

- Being prevented from doing normal work (job, housework because of the trouble)
- Hospitalization due to pain
- Change of duties/ responsibilities within the job due to pain
- An assessment by a health professional, physiotherapist, chiropractor or other such people
- Taking prescribed medication or sick leaves

In your: (Neck, Shoulder/s, Upper Back, Elbow/s, Wrist/s, Lower Back, Hip/s, Ankle/Feet)

N.B Considerations should not include disorders caused by slips, falls, motor vehicle accidents, or other similar incidents anywhere.

***Section six:*** *Questions to WMSD*

| **No.** | **Body region** | **Have you had trouble during the last 12 months?** |
| --- | --- | --- |
| **601** | Neck | 1. No 1. Yes |
| **602** | Shoulder (Both/Either) | 1. No 1. Yes |
| **603** | Upper Back | 1. No 1. Yes |
| **604** | Elbows(Both/Either) | 1. No 1. Yes |
| **605** | Wrist/Hands (Both/Either) | 1. No 1. Yes |
| **606** | Lower Back | 1. No 1. Yes |
| **607** | Hips/Thighs/Buttocks (Both/Either) | 0.No 1. Yes |
| **608** | Knees (Both/Either) | 0.No 1. Yes |
| **609** | Ankle/Feet (Both/Either) | 0.No 1. Yes |
