# Supplementary material for: Workplace stress and associated factors among vehicle repair workers in Hawassa City, Southern Ethiopia
Source: PLoS One. 2021 Apr 5;16(4):e0249640. doi: 10.1371/journal.pone.0249640 (PMC8021151; doi:10.1371/journal.pone.0249640)
Supplement: Attachment — Submitted filename: Response to the reviewers.docx [file pone.0249640.s003.docx]

**Point by point response to the reviewers**

We would like to thank the reviewers for the time and effort put on to revise our manuscript `` Prevalence of self-reported workplace stress and associated factors among vehicle repair workers in Hawassa City, South Ethiopia`` in detail. We believe that the comments have identified important areas that required improvement. We have revised in detail as PLOS One format. In addition, we did revision based on the editor`s comments and incorporated new information than what you commented. After completion of the suggested edits, the revised manuscript has benefitted from an improvement in the overall presentation and clarity.

| **Comments (****issues raised)** | **Response** |
| --- | --- |
|  | Dear Reviewers thank you for your comment and support through all processes. We included new variables that we obtained after recategorized variables from the data set (SPSS). The following points are the response to your idea. |
| 1. **Abstract**  - Abstract: - The tools used to measure workplace stress and categories of independent variables should be clearly stated. | **Accepted the comment:** Thank you for the critical observation.  I have included the following information.  Questionnaires were administered using interviews. Additional tools were used for weight and height measurements. |
| **2.Method section** | Dear Reviewer: It is right and I accepted the comment. I have described the issues in the manuscript document. |
| 2.3 study variables:  BMI and WMSD were listed as independent variables, while musculoskeletal symptoms were listed in line 117-118 with other outcome variables. It is unclear which variables were treated as dependent and which were independent variables  3.3 Job stress and 3.5 job satisfaction: Please briefly describe how the cutoff point was chosen or validated in previous literature. | Both body mass index (BMI) and work-related musculoskeletal disorders (WMSD) were independent variables. Weight in kilogram and height in centimeter were measured. BMI was calculated in kilogram per meter square (kg/m2). A digital weight scale was used for assessing weight, and height.  WMSD symptoms were measured in nine body regions using the extended version of the Nordic Musculoskeletal Questionnaire (NMQ-E). It is a valid, repeatable, sensitive and useful as a screening and surveillance tool.  # Both Job stress and job satisfaction were briefly described under the data collection tool and procedure topic.  **Job stress:**  We used the American Institute of Stress Scale to measure stress. It is a questionnaire; it was designed to apply a workplace stress data collection tool to assess job stress levels of the American workforce. The tool was validated by the American Institute of Stress. It is used to assess job stress among workers in a wide range of occupations and has been used in several studies for stress level evaluation. The questionnaire consists of eight items describing how often a respondent feels toward his or her job. The scale is in the five-point Likert response format, ranging from never (scored 1) to very often (scored 5). High scores are indicative of higher levels of job stress except item numbers 6, 7, and 8 are reverse-scored. The total scores are interpreted as follows: scores of 15 and below: relatively calm and stress isn’t much of an issue, 16–20: fairly low, 21–25: moderate levels of work stress, 26–30: severe levels of work stress, and 31–40: potentially dangerous level of work stress.  **Interpretation of the current study**: Job Stress was YES if the score measured was 16-40 and NO if the result was lower than or equal to 15.  **Job satisfaction:**  We used the Generic job satisfaction scale to assess the level of satisfaction. It is a questionnaire; was developed to collect job satisfaction levels among peoples in Ontario, Canada. The tool was validated by different studies and it is also used in a wide range of occupational groups. The questionnaire consists of ten items. Each item had a 5-point Likert response from strongly agree to strongly disagree. The scores are interpreted 42-50: very high, 39-41: high, 32-38: average, 27-3l: low and 10-25: very low  **Interpretation of the current study**: Using the job satisfaction scale YES if a measured score was 32–45 and NO if the result was lower than or equal to 10–31. No result greater than 45 and less than 10 scores was found in our study. |
| 2.2 study population:  Among the 368 vehicle workers, 344 participated in the study. Were the 24 workers excluded from the study due to inadequate work duration, or diagnosis of diseases, or disagree with study participation? Please clarify. | Dear Reviewer, thank you very much. I accepted the comment and wrote the reason under the study population and result topic.  A total of 21 workers didn’t fit the eligibility criteria and three participants disagreed to participate. A total of 344 vehicle repair workers participated in this study giving a response rate of 99.1%. |
| 3.4 Work-related musculoskeletal disorders (WMSD) is measured by self-report symptoms. It is not clear what is the difference between WMSD here and the symptoms measured by NMQ-E in line 116-117, while the NMQ-E score seems not being included in the regression models. | Dear Reviewer! I accepted the comment and revised it.  WMSDs are defined as injuries or disorders of the muscles, nerves, tendons, joints, cartilage, and spinal discs that may be associated with the work environment and performance of work. And due to the disorder, workers might report pain, ache, or discomfort in nine body regions. These symptoms appear at work and often disappear during rest or exit in life long time.  We did not apply any diagnostic instrument without the screening tool of NMQ-E. All response options of the questionnaire were dichotomous (yes/no). At least one symptom on any body part of nine body region over 12 months prior to the study was considered as WMSD Yes Otherwise NO.  WMSD is described under the operational definition topic based and WMSD Yes/No response was included in the regression models and presented in Table 3. |
| **3.Result** |  |
| Table 4 and 4.4 Prevalence and factors associated with work-related stress: Please indicate what variables were included in the crude and adjusted models. Does the crude model the same as the bivariate model mentioned in 2.4 data analysis line 148-151? In addition, the outcome variable in the logistic model in table 4 was work-related stress. It is unclear whether job satisfaction and musculoskeletal symptoms were outcome variables or independent variables? Because they were explained in the 2.3 study variable section but were not seen in the logistic model. | **Accepted the comment**: Topic 2.4 data analysis and 4.4 Prevalence and factors associated with work-related stress needed clarity. We revised it to make it more understandable. The following points might answer your question.  **Method:**  The bivariate logistic regression model shows the relation between independent variables with workplace stress (outcome or dependent variable). Therefore; one independent variable was interred into bivariate logistic regression at a time; then the model gave the relationship between one independent variable and a given outcome variable. But it shows a crude.  Multivariable logistic regression model shows the relation between an independent variable with workplace stress through adjusting cofounder /other independent variables. Therefore; more than two independent variables were entered into the model; then the model gave the relationship between one independent and a given outcome variable through adjusting cofounder or other explanatory independent variables at a time in a different row of a table. But the output of the model is affected or unstable if a large number of variables were entered into the model at a time. Since there were many independent variables in the current study, each variable was analyzed using bivariate logistic regression to select candidates for multivariable logistic regression. This step was help us to reduce an excessive number of variables and an unstable estimate in the final model. Finally, all independent variables with a *p*-value below 0.2 with the dependent variable in the bivariate analysis were selected for multivariable analysis.  **Result:**  Socio-demographic variables (educational status, employment status and service year), personal related characteristics (physical exercise, type of activity engage in after leaving the work, alcohol drinking, chewed Khat and work-related musculoskeletal disorder) and work environment (health and safety-related training, type of vehicle serviced, type of vehicle workshop, inadequate number of staff, inadequate workspace, a heavy loads job more than 20kg routinely and work posture) were fitted and selected for the multivariable logistic regression model. Service year [AOR: 2.40; 95% CI (1.29-4.50)], work-related musculoskeletal disorder [AOR: 3.39; 95% CI (1.99-5.78)], serviced large-sized vehicle [AOR: 2.03; 95% CI (1.16-3.54)] and working in squatting and lying work posture [AOR: 4.63; 95% CI (1.61-13.3)] were found to be associated with a higher likelihood of work-related stress (Table 3).  Both job satisfaction and Musculoskeletal symptoms were explained as Work-related musculoskeletal disorder (WMSD) were independent variables. Job satisfaction was not selected for Multivariable logistic regression because of p- value>0.2 in bivariate logistic regression. According to the operational definition, a single musculoskeletal symptom count as WMSD yes. Therefore; musculoskeletal symptoms were found in the logistic regression in named of WMSD (table 3) |
| **Discussion:** |  |
| 4. Discussion:  The authors compared the prevalence of work stress in line 237-246. However, it is unclear whether work stress was measured by the same tool or questionnaire in these cited studies. If not, were the prevalence rates in these studies comparable?  In the last section of discussion (line 270-274), another limitation is that work psychosocial factors were not assessed using validated tools in the literature, such as job demand-control model, and effort-reward imbalance model. This study chose work stress as the outcome but missed psychosocial work factors as risk factors. | **Accepted the comment**: I have tried to find a similar study and used a similar tool. The studies, what we found based on similar tools were used to comparing. But it was difficult to find both similar study and the same tool. So that; we included other stress studies but the inconsistency was explained in the discussion part.  . |
