# Supplementary material for: Workplace stress and associated factors among vehicle repair workers in Hawassa City, Southern Ethiopia
Source: PLoS One. 2021 Apr 5;16(4):e0249640. doi: 10.1371/journal.pone.0249640 (PMC8021151; doi:10.1371/journal.pone.0249640)
Supplement: Attachment — Submitted filename: Response to the reviewers.docx [file pone.0249640.s004.docx]

**Point by point response to the reviewers**

We would like to thank the reviewers for the time and effort put on to revise our manuscript `` Prevalence of self-reported workplace stress and associated factors among vehicle repair workers in Hawassa City, South Ethiopia`` in detail. We accepted your two issue and did revision. Also; reference editing was done on spelling and writing the missed words during manuscript writing. In addition, English language professional fixed the language related faults in the document.
